# Trends in hepatocellular carcinoma research from 2008 to 2017: a bibliometric analysis

**DOI:** 10.7717/peerj.5477

**Published:** 2018-08-15

**Authors:** Yan Miao, Ying Zhang, Lihong Yin

**Affiliations:** Key Laboratory of Environmental Medicine Engineering of Ministry of Education, School of Public Health, Southeast University, Nanjing, China

**Keywords:** Hepatocellular carcinoma, Bibliometrics, CiteSpace IV, WoSCC

## Abstract

**Objectives:**

To comprehensively analyse the global scientific outputs of hepatocellular carcinoma (HCC) research.

**Methods:**

Data of publications were downloaded from the Web of Science Core Collection. We used CiteSpace IV and Excel 2016 to analyse literature information, including journals, countries/regions, institutes, authors, citation reports and research frontiers.

**Results:**

Until March 31, 2018, a total of 24,331 papers in HCC research were identified as published between 2008 and 2017. *Oncotarget* published the most papers. China contributed the most publications and the United States occupied leading positions in H-index value and the number of ESI top papers. Llovet JM owned the highest co-citations. The keyword “transarterial chemoembolization” ranked first in the research front-line.

**Conclusions:**

The amount of papers published in HCC research has kept increasing since 2008. China showed vast progress in HCC research, but the United States was still the dominant country. Transarterial chemoembolization, epithelial-mesenchymal transition, and cancer stem cell were the latest research frontiers and should be paid more attention.

## Introduction

Hepatocellular carcinoma (HCC) is one of the most common type of primary liver malignancy and ranks third in the world’s leading causes of cancer death (Balogh et al., 2016). The highest incidence rates of HCC worldwide are in East Asia and sub-Saharan Africa, with over 20 per 100,000 individuals (Ghouri, Mian & Rowe, 2017; Zhu et al., 2016). According to data from the Surveillance, Epidemiology, and End Results (SEER) program in the United States, HCC incidence remains relatively low compared to other primary cancers (Mittal & El-Serag, 2013). However, the incidence rate has risen nearly fivefold from 1.4 to 6.7 per 100,000 individuals over the past decades (El-Serag & Kanwal, 2014; White et al., 2017). Regarding gender, HCC prevalence worldwide in males is higher than in females. The sex ratio in HCC varies from 4:1 to 2:1, depending on geographical area (Hefaiedh et al., 2013; Massarweh & El-Serag, 2017). Due to its extensive prevalence, HCC puts a substantial economic burden on the public health and medical system, whether in developed or developing countries.

Academic journals have published a large number of papers in HCC research since the past decade. However, no attempts have been made to analyse the data on publications systematically. Bibliometric analysis is defined as a quantitative analysis combining mathematical and statistical methods (Pritchard, 1969), and is a good choice for assessing trends in research activites (Dalpé, 2002). Moreover, bibliometric analysis focuses on the metrological characteristics of research literatures within a certain field (Ellegaard & Wallin, 2015), which helps investigators to grasp the development characteristics in this field over time and guide their follow-up work. In recent years, an increasing number of bibliometric studies have been published in high-impact medical journals (Aggarwal et al., 2016; Almeida-Guerrero et al., 2018; Azer, 2015; Baek et al., 2018; Bruggmann et al., 2017; Khan et al., 2018). Journals have also gradually shifted from publishing only conventional research to including bibliometric research (Wakeling et al., 2017).

The present study systematically evaluated HCC research from 2008 to 2017. We aimed to identify the mode of publications, construct research collaboration networks, and assess research trends and frontiers by time.

## Materials & Methods

### Data source and search strategy

Literature retrieval was done online through the Science Citation Index-Expanded (SCI-E) of the Web of Science Core Collection (WoSCC) on March 31, 2018. All searches were done within the same day, to avoid the bias caused by the daily database updates. The search terms were used as follows: = (“hepatocellular carcinoma*”) OR = (“hepatic cell carcinoma*”) OR = (“liver cell carcinoma*”) OR = (“liver carcinoma rupture”) OR = (“primary liver cancer*”) OR = (“primary liver carcinoma*”) OR = (“malignant hepatoma*”) OR = (“hepatocarcinoma*”) OR = (“hepatoma*”) AND Language= English. In the present study, only original and review papers were included.

### Data collection

Raw data from WoSCC were initially downloaded and verified by two authors (YZ and YM) independently. The data were then imported into Excel 2016 (Redmond, WA, USA) and CiteSpace IV (Drexel University, Philadelphia, PA, USA), and systematically analysed. Any differences were unified by discussion.

### Statistical methods

The WoSCC literature analysis report was used to analyse publication characteristics, including countries/regions, institutes, authors, journal sources, citation counts, number of annual publications, impact factor and H-index. The impact factor is an indicator that reflects the average number of yearly citations for recent papers published in the journal (Garfield, 2006). It is used to assess the quantity of research output in most bibliometric studies. H-index is a measure calculating both the productivity and citation impact per publication of a country, institute, scholar, and so forth (Costas & Bordons, 2007). It usually serves as an indicator for assessing the quality of scientific output.

Excel 2016 was used to analyse the publication trend. The polynomial model $f \left( x \right) =a{x}^{3}+b{x}^{2}+cx+d$ was applied to forecast the growth of publications in the following year. Variable *x* stands for the publication year and $f \left( x \right) $ stands for the number of publications.

CiteSpace IV was used to analyse the association between journals, explore collaboration networks between authors/institutes/countries, identify co-cited authors/references, capture keywords with strong citation bursts, and construct visualization maps of all items mentioned above. In the present study, the individual network was derived from the 50 most highly cited papers in a one-year slice (Chen, 2004). Moreover, we used the TF-IDF weighting to analyse the contents of each cluster. TF-IDF, an abbreviation of term frequency-inverse document frequency, is a statistical algorithm reflecting how significant a word to a corpus of documents (Ramos, 2003).

## Results

### Annual publications and growth forecast

**Figure 1:**
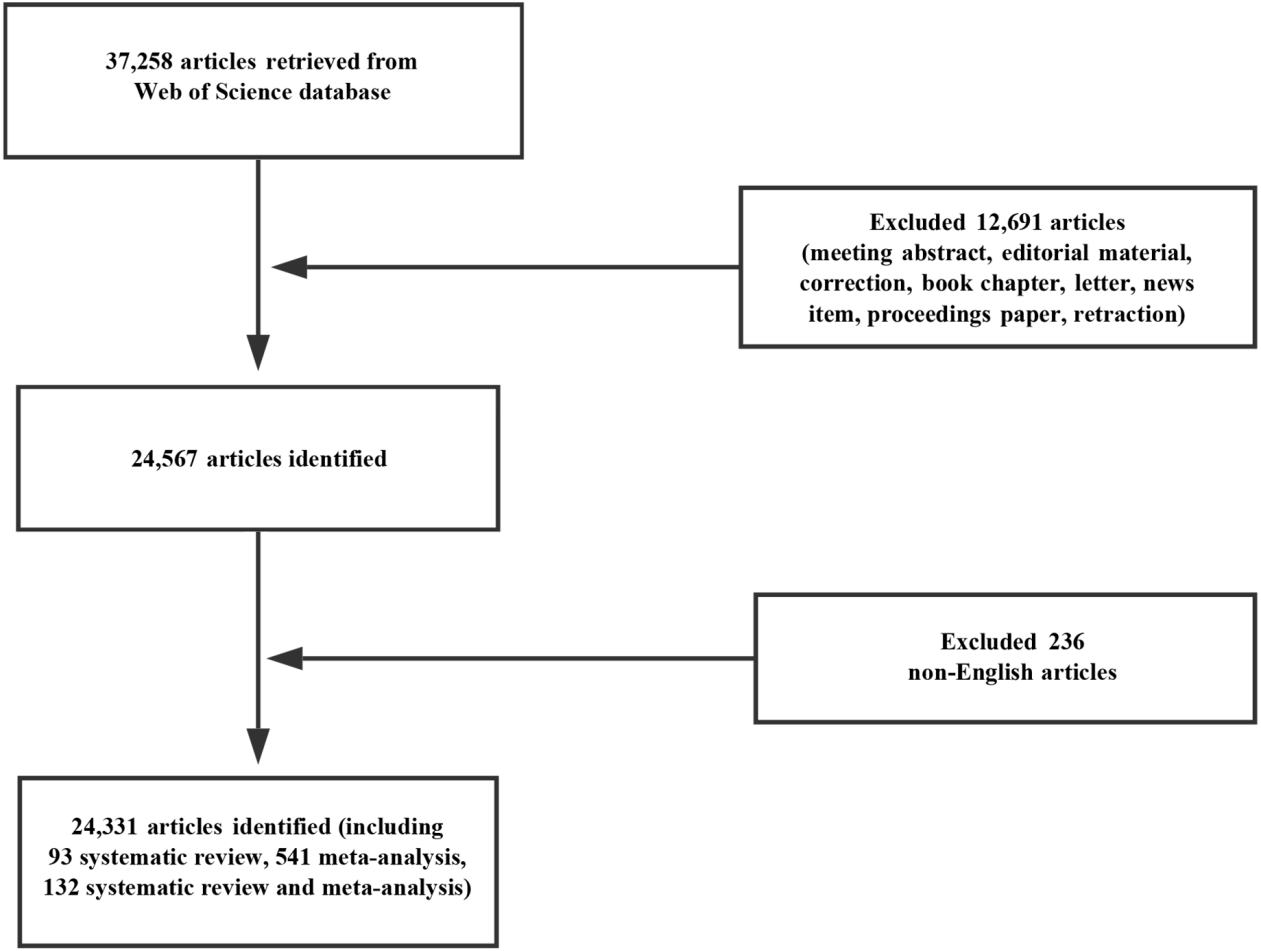
Flow chart of hepatocellular carcinoma research inclusion.

**Figure 2:**
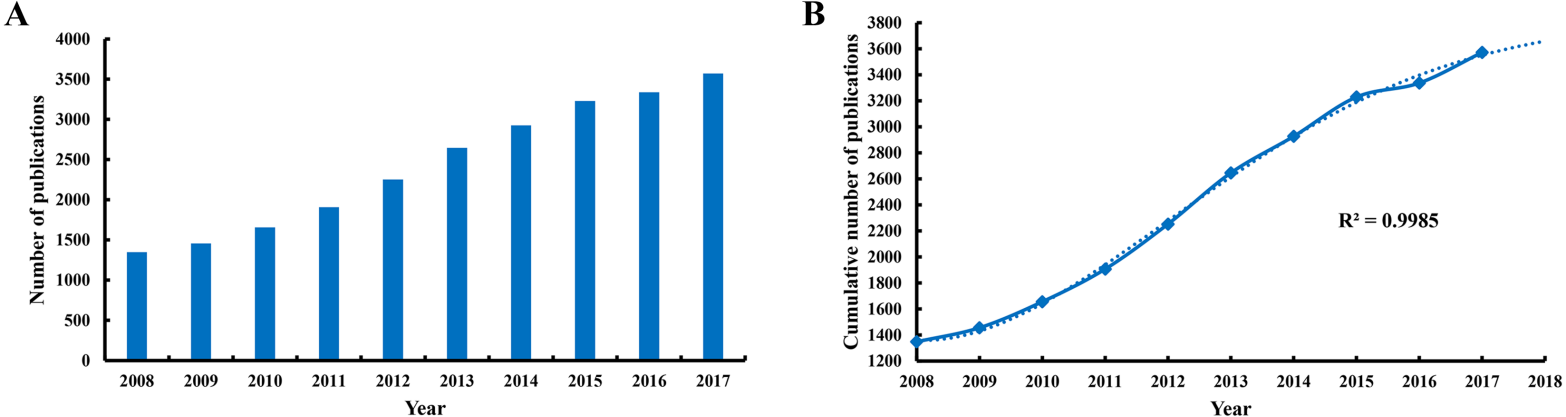
Publication outputs and growth forecast. (A) The annual number of publication in hepatocellular carcinoma research from 2008 to 2017. (B) The polynomial curve fitting of publication growth in hepatocellular carcinoma research.

In total, 24,331 papers (Fig. 1, Fig. S1) matched the retrieval criteria, including 93 systematic reviews, 541 meta-analysis, and 132 systematic review and meta-analysis. The number of publications by year was presented in Fig. 2A, where the overall trend consistently kept rising from 1,348 articles in 2008 to 3,572 articles in 2017.

The polynomial curve fitting of publication growth in HCC research showed a significant correlation (the coefficient of determination (*R*^2^) = 0.9985) between publication year and number of publications (Fig. 2B). Through curve fitting, the number of publications was estimated to reach 3,660 in 2018.

### Distribution of journals

In total, 1,681 academic journals (Dataset S1) have published papers in HCC research. Table 1 presented the top 15 journals contributing to HCC research. *Oncotarget* (impact factor (IF) 2016 = 5.168), published the most papers (852 publications, 3.50%), followed by *PLOS ONE* (IF 2016 = 2.806; 794 publications; 3.26%), *World Journal of Gastroenterology* (IF 2016 = 3.365; 705 publications; 2.90%), and *Hepatology* (IF 2016 = 13.246; 500 publications; 2.06%).

**Table 1:** The top 15 journals that published articles in hepatocellular carcinoma research.

**Rank**	**Journal title**	**Country**	**Count**	**Percent**	**IF 2016**
1	Oncotarget	United States	852	3.50	5.168
2	PLOS One	United States	794	3.26	2.806
3	World Journal of Gastroenterology	United States	705	2.90	3.365
4	Hepatology	United States	500	2.06	13.246
5	Oncology Reports	Greece	423	1.74	2.662
6	Tumor Biology	England	413	1.70	3.650
7	Hepatology Research	Japan	365	1.50	2.602
8	Oncology Letters	Greece	351	1.44	1.390
9	Journal of Hepatology	Netherlands	327	1.34	12.486
10	Hepato-Gastroenterology	Germany	283	1.16	NA
11	Journal of Gastroenterology and Hepatology	Australia	279	1.15	3.452
12	Liver International	Denmark	265	1.09	4.116
13	Cancer Letters	Netherlands	259	1.06	6.375
14	Scientific Reports	England	259	1.06	4.259
15	BMC Cancer	England	244	1.00	3.288

**Figure 3:**
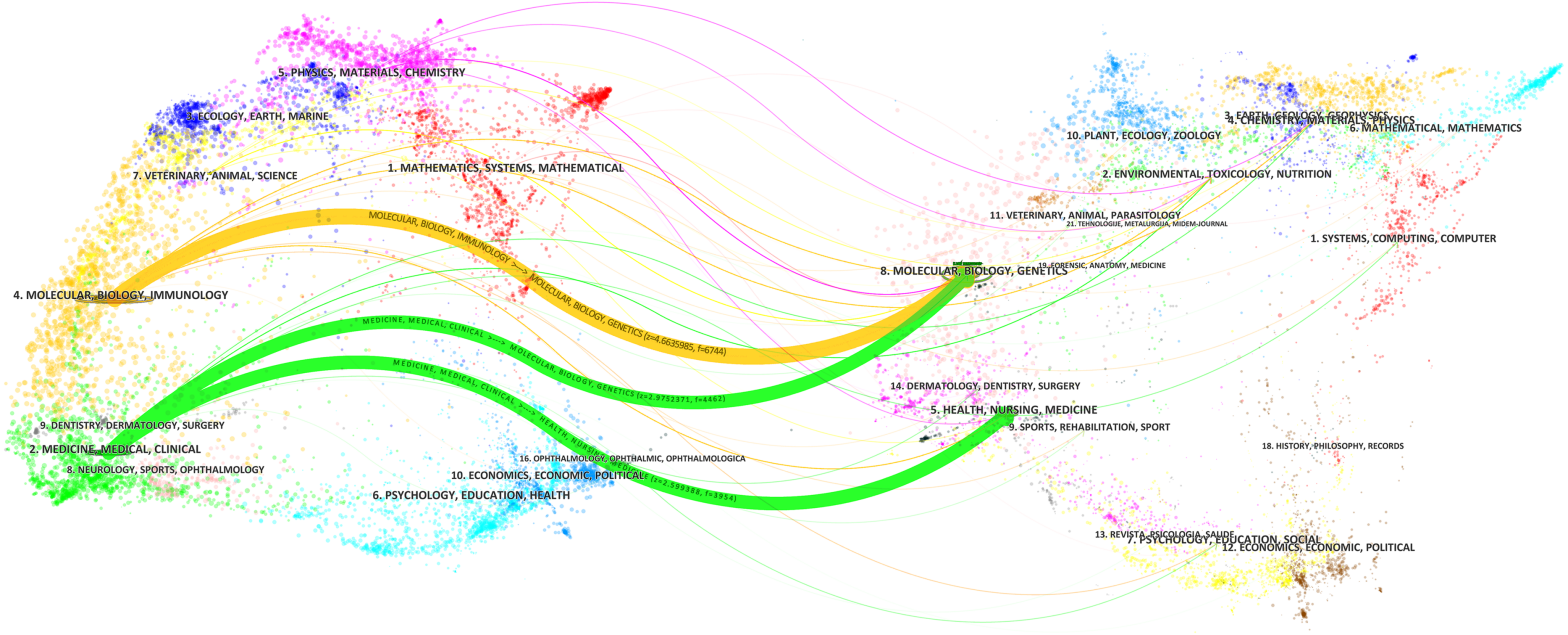
The dual-map overlay of journals related to hepatocellular carcinoma research. There were three citation paths. The yellow path, papers published in immunology/biology journals mostly cited journals in biology/genetics area; the middle green path, papers published in medical/clinical journals partially cited journals in biology/genetics area; the bottom green path, papers published in medical/clinical journals partially cited journals in health/medicine area.

Figure 3 displayed the dual-map overlay of journals. The left and right sides corresponded to the citing and cited journals maps, respectively. The labels represented the disciplines covered by the journal. The lines on the map started from the left and ended on the right, representing the citation links. There were three main citation paths shown on the map.

### Distribution of countries and institutes

**Figure 4:**
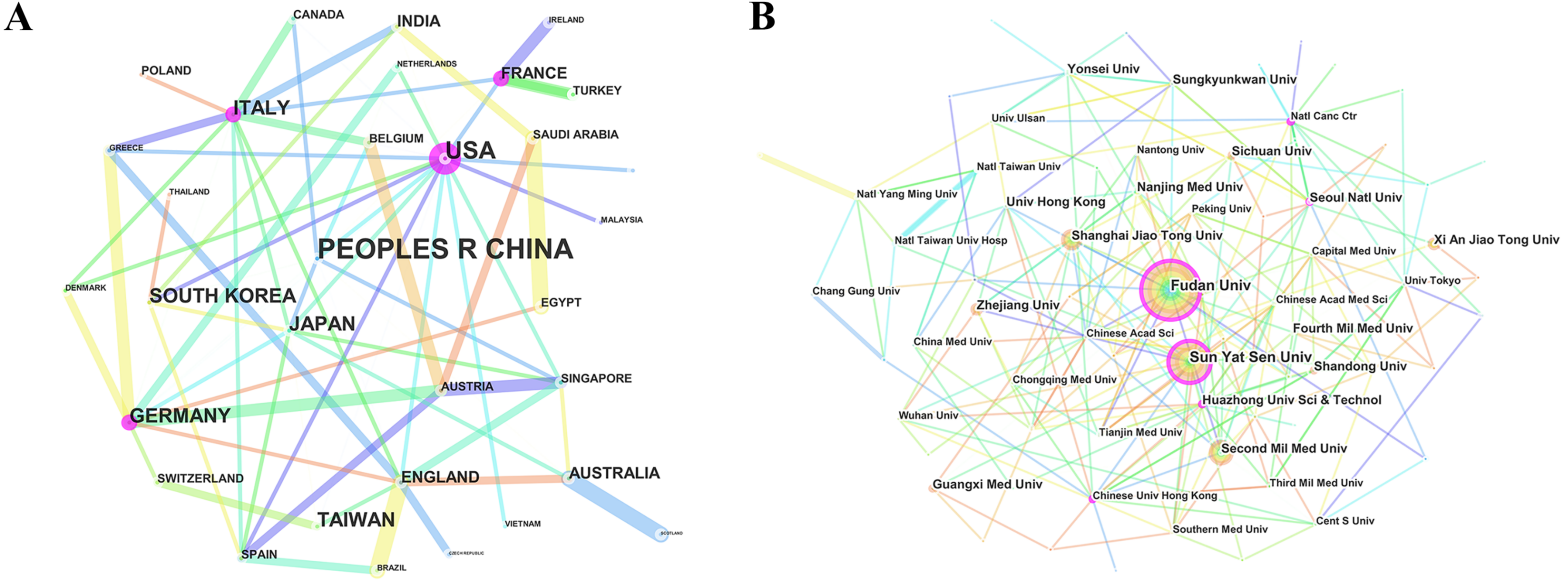
The distribution of countries and institutes. (A) The network map of countries/regions that involved in hepatocellular carcinoma research. (B) The network map of institutes that involved in hepatocellular carcinoma research.

The 24,331 papers in HCC research were contributed by 116 countries/regions (Dataset S2). Extensive collaborations were observed between countries/regions (Fig. 4A). According to the list of top 10 countries/regions (Table 2) engaged in HCC research, China contributed the most publications (10,755), followed by the United States (3,993), Japan (3,296), and South Korea (1,937).

**Table 2:** The top 10 countries and institutes contributed to publications in hepatocellular carcinoma research.

**Rank**	**Country/Region**	**Count**	**Institute**	**Count**
1	China	10,755	Fudan University	1,029
2	United States	3,993	Sun Yat-Sen University	813
3	Japan	3,296	Second Military Medical University	692
4	South Korea	1,937	Shanghai Jiao Tong University	527
5	Taiwan	1,540	Zhejiang University	475
6	Italy	1,335	University of Hong Kong	383
7	Germany	926	Seoul National University	380
8	France	729	Yonsei University	359
9	England	452	Chinese University of Hong Kong	356
10	Spain	433	Sungkyunkwan University	348

Nearly 11,000 institutes (Dataset S3) made contributions to HCC research. The collaborations between institutes were not evident (Fig. 4B), compared to countries. The top 10 institutes (Table 2) contributed more than 20% of total publications. In the list, Fudan University ranked first, followed by Sun Yat-Sen University, Second Military Medical University and Shanghai Jiao Tong University.

### Analysis of ESI top papers, H-index, and citations

**Figure 5:**
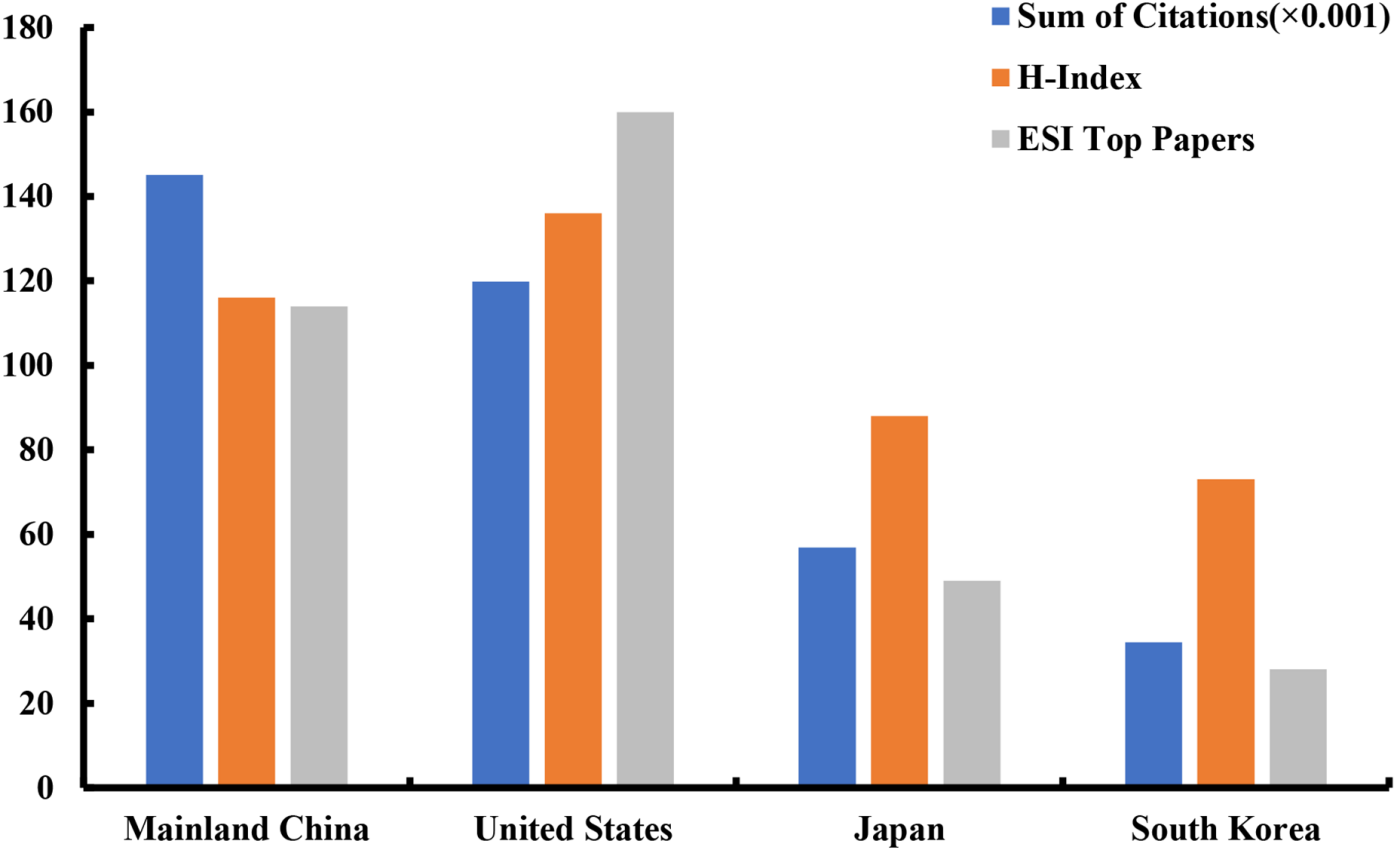
The distribution of ESI top papers, H-index, and citation (×0.001) in the top four countries.

Among the top four productive countries (Fig. 5), the United States contributed the most number of ESI top papers (160) and achieved the highest H-index value (136). Due to a vast amount of literature, China owned the most citation counts (145,060). The other two countries, Japan and South Korea, did not have advantages in the ranking of the three items mentioned above.

### Distribution of authors

**Figure 6:**
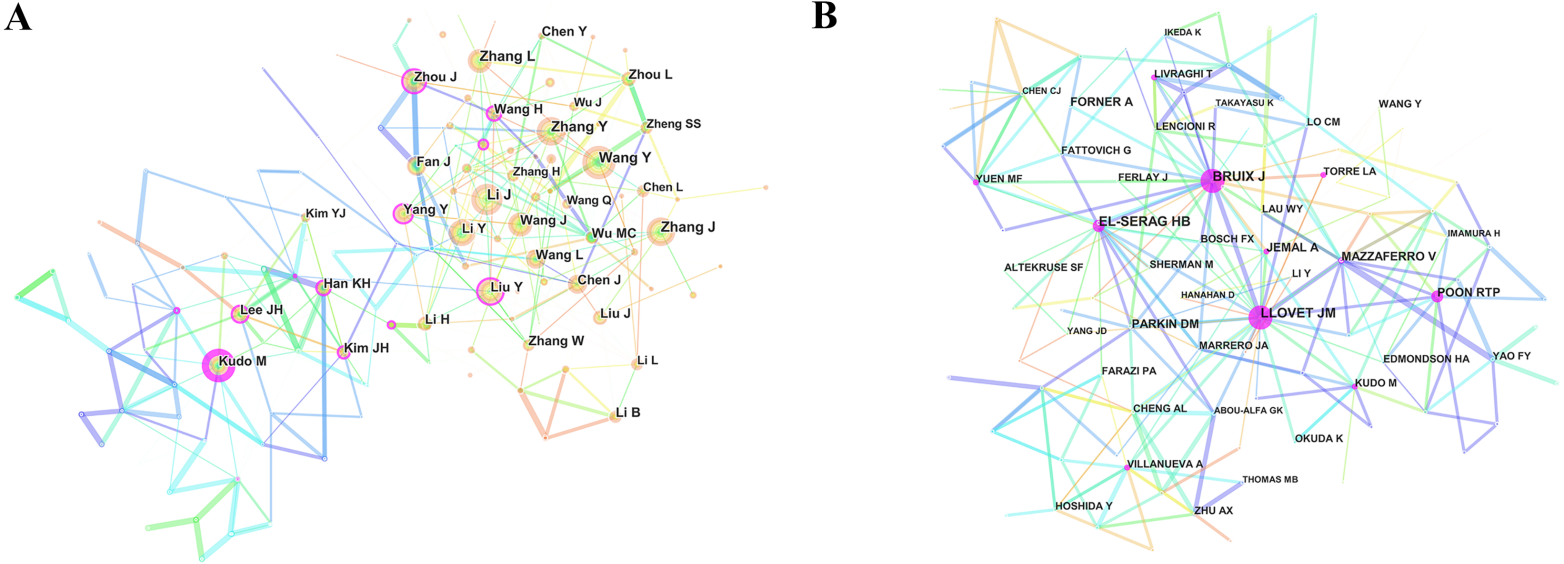
The distribution of authors. (A) The network map of active authors contributed to hepatocellular carcinoma research. (B) The network map of co-cited authors contributed to hepatocellular carcinoma research.

Over 55,900 authors (Dataset S4) contributed to HCC research. Figure 6A showed the collaboration network between authors. Among the top 10 contributive authors (Table 3), Wang Y (297 publications) was ranked first, followed by Li J (291 publications), Zhang Y (279 publications) and Zhang J (264 publications).

**Table 3:** The top 10 authors, co-cited authors, and co-cited references in hepatocellular carcinoma research.

**Rank**	**Author**	**Count**	**Co-cited Author**	**Count**	**Co-cited Reference**	**Count**
1	Wang Y	297	Llovet JM	7,351	Bruix J, 2011, Hepatology, V53, P1020	1,730
2	Li J	291	Bruix J	5,946	Jemal A, 2011, CA-Cancer J Clin, V61, P69	1,578
3	Zhang Y	279	EL-Serag HB	5,038	Llovet JM, 2012, J Hepatol, V56, P908	1,206
4	Zhang J	264	Parkin DM	2,769	Forner A, 2012, Lancet, V379, P1245	1,196
5	Zhang L	254	Jemal A	2,363	Llovet JM, 2008, New Engl J Med, V359, P378	1,193
6	Li Y	240	Poon RTP	2,042	Cheng AL, 2009, Lancet Oncol, V10, P25	855
7	Fan J	237	Forner A	2,010	EL-Serag HB, 2011, New Engl J Med, V365, P1118	825
8	Wang J	235	Mazaferro V	1,950	Torre LA, 2015, CA-Cancer J Clin, V65, P87	721
9	Liu Y	226	Cheng AL	1,614	EL-Serag HB, 2007, Gastroenterology, V132, P2557	674
10	Zhou J	225	Lencioni R	1,559	EL-Serag HB, 2012, Gastroenterology, V142, P1264	521

CiteSpace analysed the information of author citations and visualized it in a co-citation network (Fig. 6B). Among the top 10 co-cited authors (Table 3, Fig. S2), Llovet JM (7,351 co-citations) was ranked first, followed by Bruix J (5,946 co-citations), EL-Serag HB (5,038 co-citations) and Parkin DM (2,769 co-citations).

### Analysis of references

**Figure 7:**
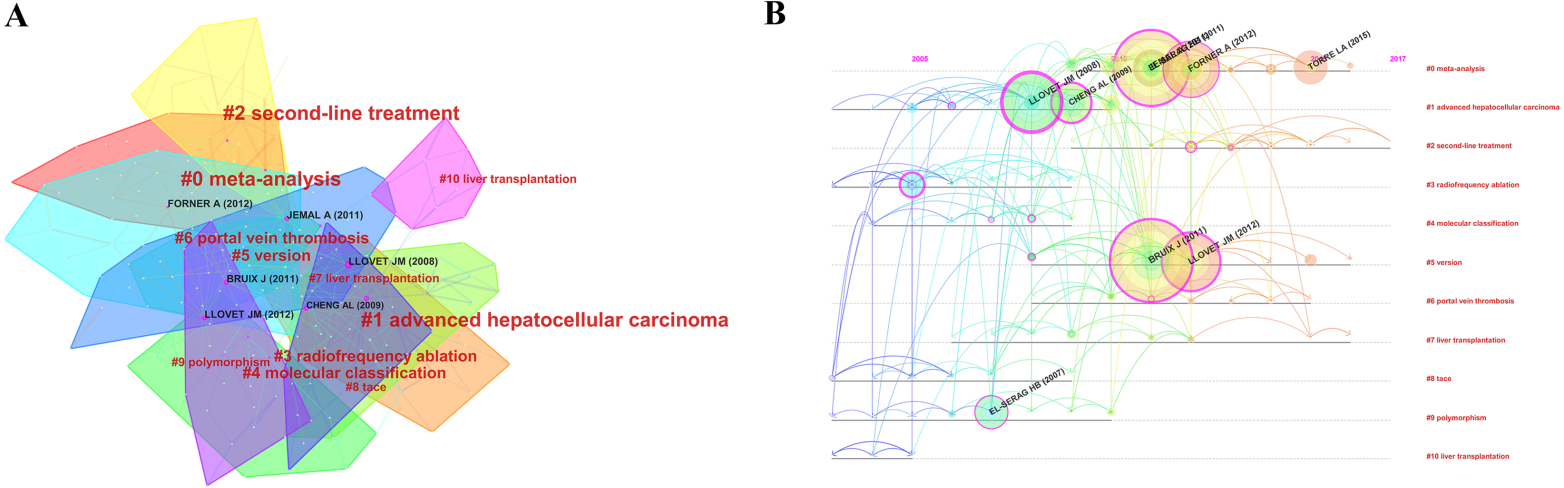
The analysis of references. (A) The co-citation map of references from publications in hepatocellular carcinoma research. (B) The timeline view of co-cited references from publications in hepatocellular carcinoma research.

We used CiteSpace IV to construct a network of co-cited references (Fig. 7A) that revealed the relevance between papers. The values of Modularity Q and Mean Silhouette both were more than 0.5 (Fig. S3), indicating that the distributivity and homogeneity of clusters were reasonable and acceptable. All clusters were named after terms extracted from the references of publications (Fig. S4). In this network, the first massive cluster was named “#0 meta-analysis”, followed by the second, named “#1 advanced hepatocellular carcinoma”, and the third, named “#2 second-line treatment.” Furthermore, the timeline view of these clusters was shown in Fig. 7B.

### Analysis of burst keywords

**Figure 8:**
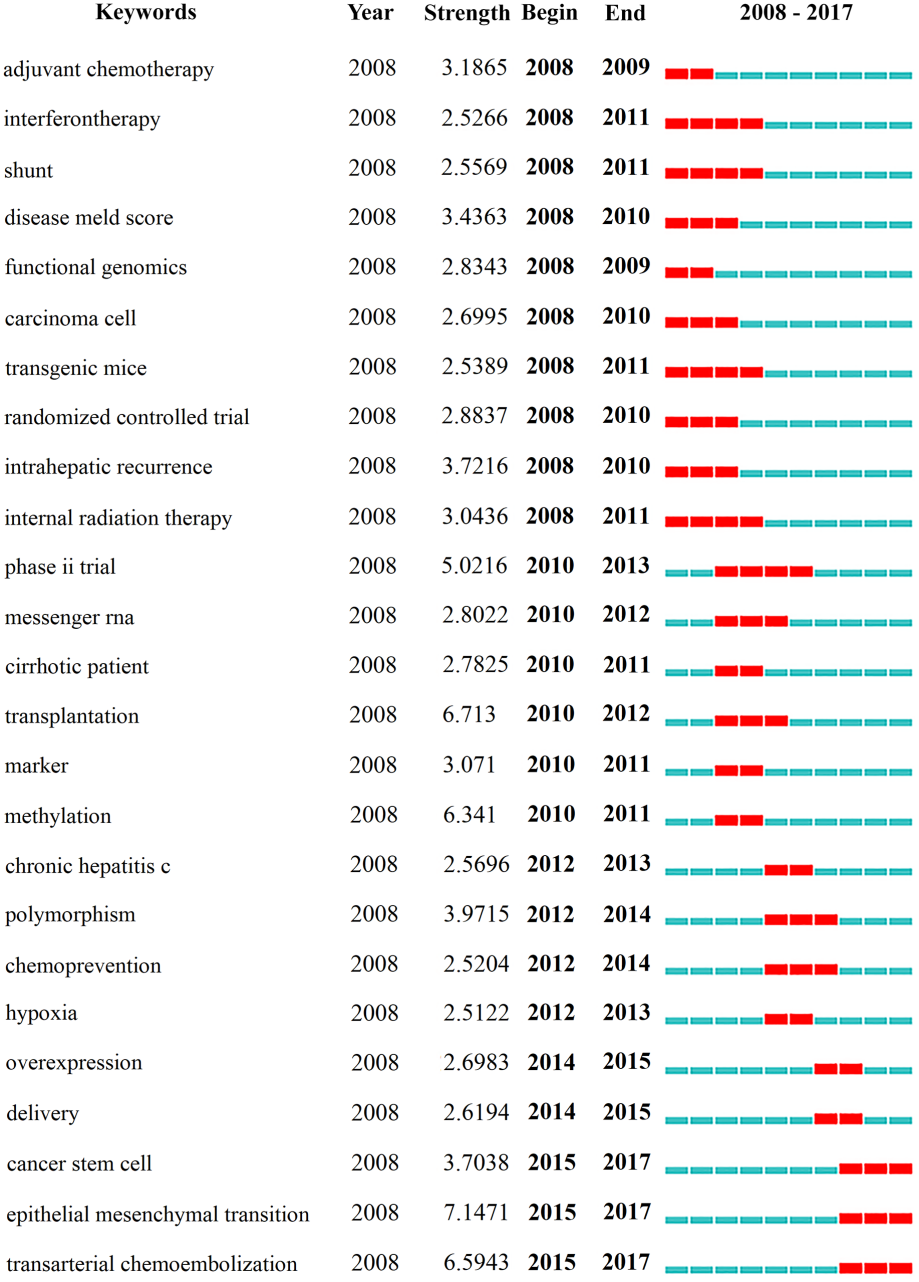
The keywords with the strong citation bursts in articles related to hepatocellular carcinoma research published from 2008 to 2017.

We identified keywords with strong citation bursts through CiteSpace IV (Fig. 8, Fig. S5). Among them, the keywords that had citation bursts after 2014 were listed as follows: “overexpression” (2014–2015), “delivery” (2014–2015), “cancer stem cell” (2015–2017), “epithelial-mesenchymal transition” (2015–2017), and “transarterial chemoembolization” (2015–2017).

## Discussion

### General information

At the beginning of the study, we searched for HCC-related papers published from 2000 to 2017 in the Web of Science. The overall trend of publications between 2000 and 2007 was stable, with only slowly increasing (Fig. S6). The total number of publications during this period was relatively small, compared with the later period (2008–2017). For this study, we were committed to identify a trend that has obvious changes in the number of publications. Besides to including a sufficient number of articles, the time span cannot be too long, either, we finally determined to limit the search from 2008 onwards. In relation to the top 15 academic journals, two journals, *Hepatology* (IF 2016 = 13.246) and *Journal of Hepatology* (IF 2016 = 12.486) had an IF higher than 10; two others, *Oncotarget* (IF 2016 = 5.168) and *Cancer Letters* (IF 2016 = 6.375) had an IF between 10 and five; five journals, *World Journal of Gastroenterology* (IF 2016 = 3.365), *Tumor Biology* (IF 2016 = 3.650), *Journal of Gastroenterology and Hepatology* (IF 2016 = 3.452), *Liver International* (IF 2016 = 4.116), and *BMC Cancer* (IF 2016 = 3.288) had an IF between five and three. Furthermore, academic journals with a high IF (higher than three) accounted for 16.86% (*N* = 4,103) of total publications.

Among the top 10 contributive countries/regions in HCC research, China was the only one from the developing world, showing its vast progress in life science over the past decade. China had an absolute advantage in the number of papers published, which also received a large number of citations. However, the United States occupied the first positions in both ESI top papers and H-index. In terms of research quality, the United States was the dominant country in HCC research. The most active collaborations were observed between Saudi Arabia and Egypt, Australia and Scotland and England and Brazil. Moreover, the collaborations among European countries were much stronger than those among Asian countries.

The top 10 institutes contributed to 5,362 papers, which accounted for 22.03% of total publications. In this list, the top five institutes were all from China. Additionally, there were 3,994 Chinese institutes involved in HCC research (Dataset S3), accounting for 36.34% of the total number of research institutes worldwide. According to recent reports, China accounted for more than half of world’s HCC patients, including over 466,000 new cases in 2015 (Zhu et al., 2016). The estimated incidence rate of HCC was 30.62 per 100,000 standard population, resulting it as the second common malignancy in China (Tanaka et al., 2011). Except for that, there are limited treatment options for unresectable HCC patients and the overall prognosis of HCC is very poor (Xie et al., 2017), making it a serious health issue in current China. That is the reason why a considerable number of Chinese institutes engaged in HCC research and China leads in the number of papers published.

### Citation information

Among the top 10 active authors, each person has published at least 225 papers; they were regarded as prolific authors. Despite that, none of them are included as top co-cited authors, suggesting that prolific authors should focus not only on number of publications but also quality of research. Regarding co-cited authors, those with more than 5,000 co-citations, including Llovet JM, who discovered the critical role of mTOR signaling in HCC pathogenesis (Villanueva et al., 2008), Bruix J, who provided guidelines for HCC management (Bruix & Sherman, 2011) and EL-Serag HB, who elaborated the epidemiology of HCC and viral hepatitis (El-Serag, 2012), have made significant contributions in this field.

The co-citation clusters in the timeline view demonstrated that top co-cited references were mainly gathered between 2008 and 2012. Meanwhile, among the top 100 co-cited references identified by CiteSpace IV, there were 72 items existed in the period from 2008 to 2012 (Fig. S4). Given this result, the period (2008–2012) could be considered as a “golden phase” of HCC research within the past decade. Table 3 presented the top 10 co-cited references in HCC research. Bruix J (2011), who published a paper in *Hepatology*, had the highest co-citations (1730), followed by Jemal A (2011, 1,578 co-citations), Llovet JM (2012, 1,206 co-citations) and Forner A (2012, 1,196 co-citations), who published papers in *CA: A Cancer Journal for Clinicians*, *Journal of Hepatology* and *The Lancet* respectively. Additionally, other journals with highest impact factor have also contributed some papers on HCC research during the past decade (Dataset S1), such as *New England Journal of Medicine* (5 papers), *The Lancet* (12 papers), *Nature* (10 papers), and *Cell* (nine papers). They were the fundamentals of this field.

### Research frontiers

We used CiteSpace IV to capture the burst keywords, which could be considered a prediction of research frontiers. As shown in Fig. 8, the blue line represented the time intervals and the red line represented the period of citation bursts. Here, we listed three frontiers of HCC research as follows:

i. Transarterial chemoembolization: Transarterial chemoembolization (TACE) is considered as an effective treatment for intermediate or advanced HCC patients (Han & Kim, 2015). In the United States, TACE is not only the most common therapy for HCC patients but also the most common bridging therapy for patients waitlisted for liver transplantation (Shah et al., 2011; Thuluvath et al., 2010). According to a retrospective study of the SEER database, TACE utilization significantly improved survival for HCC patients, especially those at an intermediate stage (Gray et al., 2017). Additionally, a recent study has proved that TACE can be a useful treatment option for HCC patients with segmental portal vein tumour thrombus (Choi et al., 2017).
ii. Epithelial-mesenchymal transition: Epithelial-mesenchymal transition (EMT) is a biological process in which epithelial cells gradually change into a mesenchymal-like type (Van Zijl et al., 2009). This process has proved to be involved in various pathological conditions, including inflammation, fibrosis and cancer (Barriere et al., 2015; Skrypek et al., 2017). Increasing evidence demonstrated that EMT plays a vital role in transferring malignant hepatocytes during the progression of HCC (Giannelli et al., 2016; Huaman et al., 2018; Nitta et al., 2008). The association between EMT and HCC raises a demand to exploit novel diagnostic and therapeutic strategies against HCC progression.
iii. Cancer stem cell: Solid tumours contain a small fraction of tumorigenic cells, known as cancer stem cells (CSCs) (Valent et al., 2012). CSCs play a crucial role in tumour metastasis/recurrence and have been identified in many malignant tumours, including HCC (Valent et al., 2012). Accumulating studies have illustrated that HCC CSCs could be enriched by several different markers, including CD133, CD90, CD24, CD13 and EpCAM (Feng et al., 2014; Kim & Park, 2014; Sainz Jr & Heeschen, 0000). HCC CSCs could partially explain the heterogeneity of HCC, metastasis after hepatic tumour resection and chemotherapeutic resistance in advanced HCC cells (Ji & Wang, 2012; Zheng et al., 2018), which provide the potential to develop novel therapeutic strategies based on stem cell biology.

### Strengths and limitations

To the best of our understanding, this paper is the first bibliometric analysis on HCC research trend over the past decade. The data analysis process was relatively objective. However, most publications retrieved from the database were written in English, causing incomplete analysis to some extent. Furthermore, this study consisted exclusively of original and review articles published between 2008 and 2017 and indexed by the Web of Science. It may not be enough to represent all HCC literature, such as other document types published in journals, books, and conferences were not included. The analysis in this study was based on articles recorded in the Science Citation Index-Expanded (SCI-E) of the Web of Science Core Collection (WoSCC). Each journal to which the SCI-E articles belong had its corresponding citation report provided by the Web of Science. Although other databases such as PubMed, Scopus, and Embase could provide a broader range of coverage, much of the “extra coverage” could be attributed to journals with potentially limited readers. Given that our objective was to conduct a high-quality bibliometric analysis to identify research trends in the core of HCC field, the SCI-E articles from WoSCC may be the only appropriate choice. Therefore, the results from other databases were not included. As for China ranked first in publications, except for the active participation of Chinese institutes, the strong support of science funding in China could be another important reason. A recent study has shown that the proportion of funded papers in China is the highest, compared to other countries. Nearly 80% of SCI-E papers are supported by science funding (Sun et al., 2013). Therefore, the advantage of the number of publications from China is particularly prominent, which may create an illusion that the gap between western countries and China is widening. Finally, although the Web of Science database is still updating, this study covers the vast majority of papers in HCC research since 2008; new data may not influence the final results.

## Conclusions

The number of publications in HCC research has been increasing over the past decade. The United States, Japan, and China were the top three countries contributing to HCC studies. There were active collaborations between developed countries. Although many Chinese institutes were engaged in HCC research, the United States was still the dominant country. Llovet JM, Bruix J and EL-Serag HB may be ideal candidates for academic cooperation. Transarterial chemoembolization, epithelial-mesenchymal transition and cancer stem cell may be frontiers in this field, and researchers should pay close attention to relevant studies in the coming years.

##  Supplemental Information

10.7717/peerj.5477/supp-1 Dataset S1 Journal sources of papers related to hepatocellular carcinoma research that were extracted from the Web of Science Core Collection

10.7717/peerj.5477/supp-2 Dataset S2 Countries/territories involved in hepatocellular carcinoma research that were extracted from the Web of Science Core Collection

10.7717/peerj.5477/supp-3 Dataset S3 Institutions involved in hepatocellular carcinoma research that were extracted from the Web of Science Core Collection

10.7717/peerj.5477/supp-4 Dataset S4 Authors involved in hepatocellular carcinoma research that were extracted from the Web of Science Core Collection

10.7717/peerj.5477/supp-5 Figure S1 The search history on hepatocellular carcinoma that was extracted from the Web of Science

10.7717/peerj.5477/supp-6 Figure S2 The co-cited authors that were identified by CiteSpace IV

10.7717/peerj.5477/supp-7 Figure S3 The co-citation map of references that was generated by CiteSpace IV

10.7717/peerj.5477/supp-8 Figure S4 The co-cited references that were identified by CiteSpace IV

10.7717/peerj.5477/supp-9 Figure S5 The keywords with strong citation bursts that were identified by CiteSpace IV

10.7717/peerj.5477/supp-10 Figure S6 The number of publications by year from 2000 to 2017, extracted from the Web of Science

## Additional Information and Declarations

### Data Availability

The following information was supplied regarding data availability: Figshare: http://figshare.com/articles/Raw_data_for_Trends_in_hepatocellular_carcinoma_from_2008_to_2017_A_bibliometric_analysis_/6885119.

## References

[ref-1] Aggarwal A, Lewison G, Idir S, Peters M, Aldige C, Boerckel W, Boyle P, Trimble EL, Roe P, Sethi T, Fox J, Sullivan R (2016). The state of lung cancer research: a global analysis. Journal of Thoracic Oncology.

[ref-2] Almeida-Guerrero A, Olaya-Gomez JC, Sanchez-Ramirez N, Murillo-Garcia DR, Cardona-Ospina JA, Lagos-Grisales GJ, Rodriguez-Morales AJ (2018). Mitigation of the global impact of Lassa fever: have we investigated enough about this Arenavirus?—a bibliometric analysis of Lassa Fever research. Travel Medicine and Infectious Disease.

[ref-3] Azer SA (2015). The top-cited articles in medical education: a bibliometric analysis. Academic Medicine.

[ref-4] Baek S, Yoon DY, Lim KJ, Cho YK, Seo YL, Yun EJ (2018). The most downloaded and most cited articles in radiology journals: a comparative bibliometric analysis. European Radiology.

[ref-5] Balogh J, Victor D, Asham EH, Burroughs SG, Boktour M, Saharia A, Li X, Ghobrial RM, Monsour HP (2016). Hepatocellular carcinoma: a review. Journal of Hepatocellular Carcinoma.

[ref-6] Barriere G, Fici P, Gallerani G, Fabbri F, Rigaud M (2015). Epithelial mesenchymal transition: a double-edged sword. Clinical and Translational Medicine.

[ref-7] Bruggmann D, Pulch K, Klingelhofer D, Pearce CL, Groneberg DA (2017). Ovarian cancer: density equalizing mapping of the global research architecture. International Journal of Health Geographics.

[ref-8] Bruix J, Sherman M (2011). Management of hepatocellular carcinoma: an update. Hepatology.

[ref-9] Chen C (2004). Searching for intellectual turning points: progressive knowledge domain visualization. Proceedings of the National Academy of Sciences of the United States of America.

[ref-10] Choi JW, Kim HC, Lee JH, Yu SJ, Kim YJ, Yoon JH, Jae HJ, Hur S, Lee M, Chung JW (2017). Transarterial chemoembolization of hepatocellular carcinoma with segmental portal vein tumour thrombus. European Radiology.

[ref-11] Costas R, Bordons M (2007). The h-index: advantages, limitations and its relation with other bibliometric indicators at the micro level. Journal of Informetrics.

[ref-12] Dalpé R (2002). Bibliometric analysis of biotechnology. Scientometrics.

[ref-13] El-Serag HB (2012). Epidemiology of viral hepatitis and hepatocellular carcinoma. Gastroenterology.

[ref-14] El-Serag HB, Kanwal F (2014). Epidemiology of hepatocellular carcinoma in the United States: where are we? where do we go?. Hepatology.

[ref-15] Ellegaard O, Wallin JA (2015). The bibliometric analysis of scholarly production: how great is the impact?. Scientometrics.

[ref-16] Feng D, Wang N, Hu J, Li W (2014). Surface markers of hepatocellular cancer stem cells and their clinical potential. Neoplasma.

[ref-17] Garfield E (2006). The history and meaning of the journal impact factor. Journal of the American Medical Association.

[ref-18] Ghouri YA, Mian I, Rowe JH (2017). Review of hepatocellular carcinoma: epidemiology, etiology, and carcinogenesis. Journal of Carcinogenesis.

[ref-19] Giannelli G, Koudelkova P, Dituri F, Mikulits W (2016). Role of epithelial to mesenchymal transition in hepatocellular carcinoma. Journal of Hepatology.

[ref-20] Gray SH, White JA, Li P, Kilgore ML, Redden DT, Abdel Aal AK, Simpson HN, McGuire B, Eckhoff DE, Dubay DA (2017). A SEER database analysis of the survival advantage of transarterial chemoembolization for hepatocellular carcinoma: an underutilized therapy. Journal of Vascular and Interventional Radiology.

[ref-21] Han K, Kim JH (2015). Transarterial chemoembolization in hepatocellular carcinoma treatment: barcelona clinic liver cancer staging system. World Journal of Gastroenterology.

[ref-22] Hefaiedh R, Ennaifer R, Romdhane H, Ben Nejma H, Arfa N, Belhadj N, Gharbi L, Khalfallah T (2013). Gender difference in patients with hepatocellular carcinoma. Tunisie Medicale.

[ref-23] Huaman J, Bach C, Ilboudo A, Ogunwobi OO, Liu C (2018). Epithelial-to-mesenchymal transition in hepatocellular carcinoma. Precision molecular pathology of liver cancer.

[ref-24] Ji J, Wang XW (2012). Clinical implications of cancer stem cell biology in hepatocellular carcinoma. Seminars in Oncology.

[ref-25] Khan NR, Saad H, Oravec CS, Norrdahl SP, Fraser B, Wallace D, Lillard JC, Motiwala M, Nguyen VN, Lee SL, Jones AV, Ajmera S, Kalakoti P, Dave P, Moore KA, Akinduro O, Nyenwe E, Vaughn B, Michael LM, Klimo Jr P (2018). An analysis of publication productivity during residency for 1506 neurosurgical residents and 117 residency departments in North America. Neurosurgery.

[ref-26] Kim H, Park YN (2014). Hepatocellular carcinomas expressing ‘stemness’-related markers: clinicopathological characteristics. Digestive Diseases.

[ref-27] Massarweh NN, El-Serag HB (2017). Epidemiology of hepatocellular carcinoma and intrahepatic cholangiocarcinoma. Cancer Control.

[ref-28] Mittal S, El-Serag HB (2013). Epidemiology of HCC: consider the population. Journal of Clinical Gastroenterology.

[ref-29] Nitta T, Kim JS, Mohuczy D, Behrns KE (2008). Murine cirrhosis induces hepatocyte epithelial mesenchymal transition and alterations in survival signaling pathways. Hepatology.

[ref-30] Pritchard A (1969). Statistical bibliography or bibliometrics. Journal of Documentation.

[ref-31] Ramos J (2003). Using tf-idf to determine word relevance in document queries. http://citeseerx.ist.psu.edu/viewdoc/download?doi=10.1.1.121.1424rep=rep1type=pdf.

[ref-32] Sainz Jr B, Heeschen C Standing out from the crowd: cancer stem cells in hepatocellular carcinoma. Cancer Cell.

[ref-33] Shah SA, Smith JK, Li Y, Ng SC, Carroll JE, Tseng JF (2011). Underutilization of therapy for hepatocellular carcinoma in the medicare population. Cancer.

[ref-34] Skrypek N, Goossens S, De Smedt E, Vandamme N, Berx G (2017). Epithelial-to-Mesenchymal transition: epigenetic reprogramming driving cellular plasticity. Trends in Genetics.

[ref-35] Sun JW, Liu D, Wang XW, Hou HY (2013). Science funding and SCI papers output: a comparative analysis on 10 countries. Studies in Science of Science.

[ref-36] Tanaka M, Katayama F, Kato H, Tanaka H, Wang J, Qiao YL, Inoue M (2011). Hepatitis B and C virus infection and hepatocellular carcinoma in China: a review of epidemiology and control measures. Journal of Epidemiology.

[ref-37] Thuluvath PJ, Guidinger MK, Fung JJ, Johnson LB, Rayhill SC, Pelletier SJ (2010). Liver transplantation in the United States, 1999–2008. American Journal of Transplantation.

[ref-38] Valent P, Bonnet D, De Maria R, Lapidot T, Copland M, Melo JV, Chomienne C, Ishikawa F, Schuringa JJ, Stassi G, Huntly B, Herrmann H, Soulier J, Roesch A, Schuurhuis GJ, Wohrer S, Arock M, Zuber J, Cerny-Reiterer S, Johnsen HE, Andreeff M, Eaves C (2012). Cancer stem cell definitions and terminology: the devil is in the details. Nature Reviews Cancer.

[ref-39] Van Zijl F, Zulehner G, Petz M, Schneller D, Kornauth C, Hau M, Machat G, Grubinger M, Huber H, Mikulits W (2009). Epithelial-mesenchymal transition in hepatocellular carcinoma. Future Oncol.

[ref-40] Villanueva A, Chiang DY, Newell P, Peix J, Thung S, Alsinet C, Tovar V, Roayaie S, Minguez B, Sole M, Battiston C, Van Laarhoven S, Fiel MI, Di Feo A, Hoshida Y, Yea S, Toffanin S, Ramos A, Martignetti JA, Mazzaferro V, Bruix J, Waxman S, Schwartz M, Meyerson M, Friedman SL, Llovet JM (2008). Pivotal role of mTOR signaling in hepatocellular carcinoma. Gastroenterology.

[ref-41] Wakeling S, Willett P, Creaser C, Fry J, Pinfield S, Spezi V (2017). Transitioning from a conventional to a ‘mega’journal: a bibliometric case study of the journal medicine. Publications.

[ref-42] White DL, Thrift AP, Kanwal F, Davila J, El-Serag HB (2017). Incidence of hepatocellular carcinoma in all 50 United States, from 2000 through 2012. Gastroenterology.

[ref-43] Xie D-Y, Ren Z-G, Zhou J, Fan J, Gao Q (2017). Critical appraisal of Chinese 2017 guideline on the management of hepatocellular carcinoma. Hepatobiliary Surgery and Nutrition.

[ref-44] Zheng H, Pomyen Y, Hernandez MO, Li C, Livak F, Tang W, Dang H, Greten TF, Davis JL, Zhao Y, Mehta M, Levin Y, Shetty J, Tran B, Budhu A, Wang XW (2018). Single cell analysis reveals cancer stem cell heterogeneity in hepatocellular carcinoma. Hepatology.

[ref-45] Zhu RX, Seto W-K, Lai C-L, Yuen M-F (2016). Epidemiology of Hepatocellular Carcinoma in the Asia-Pacific Region. Gut and Liver.

