# Supplementary figures and images for: Trends in hepatocellular carcinoma research from 2008 to 2017: a bibliometric analysis
Source: PeerJ. 2018 Aug 15;6:e5477. doi: 10.7717/peerj.5477 (PMC6098682; doi:10.7717/peerj.5477)

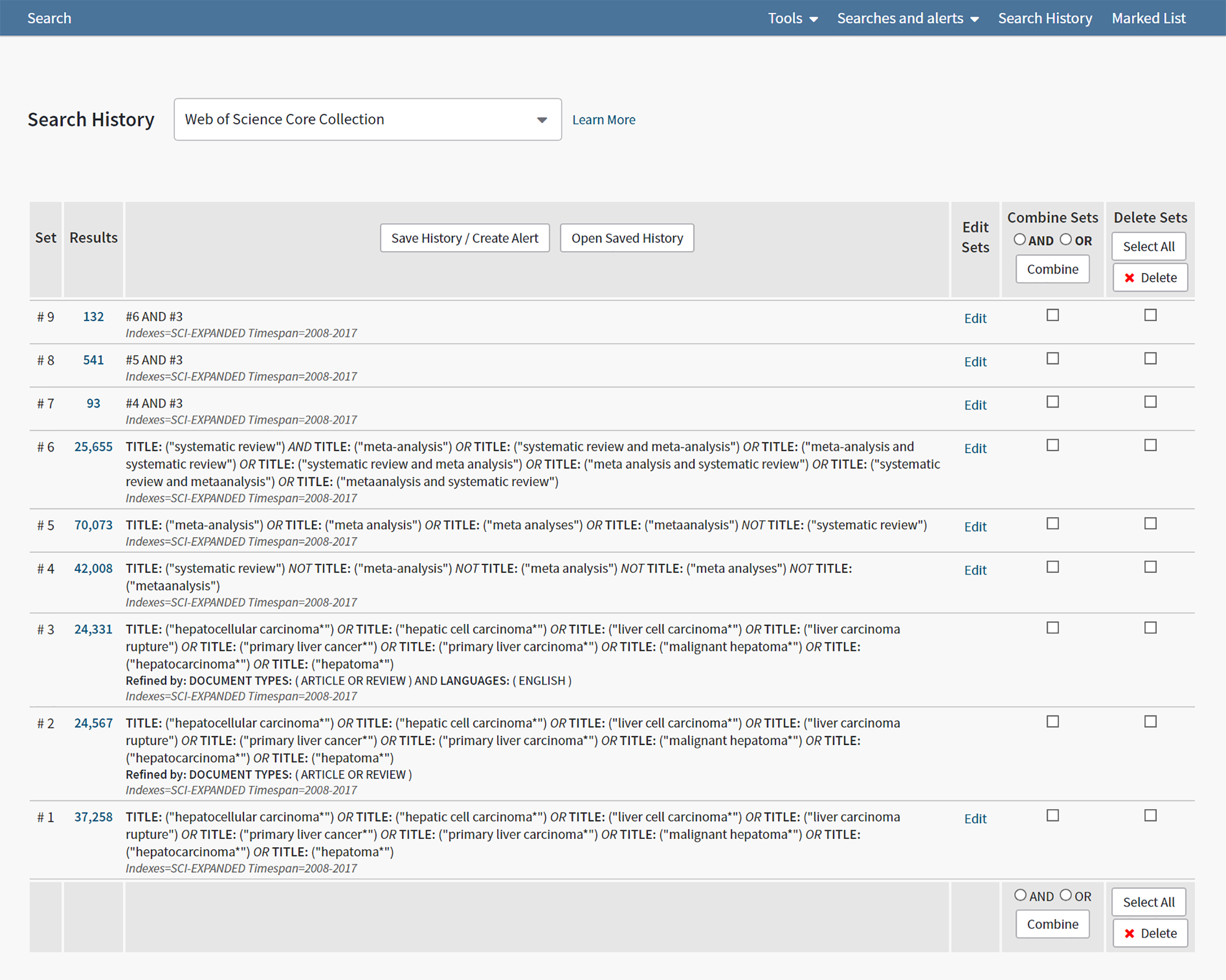

Supplement: Figure S1 [file peerj-06-5477-s005.png]

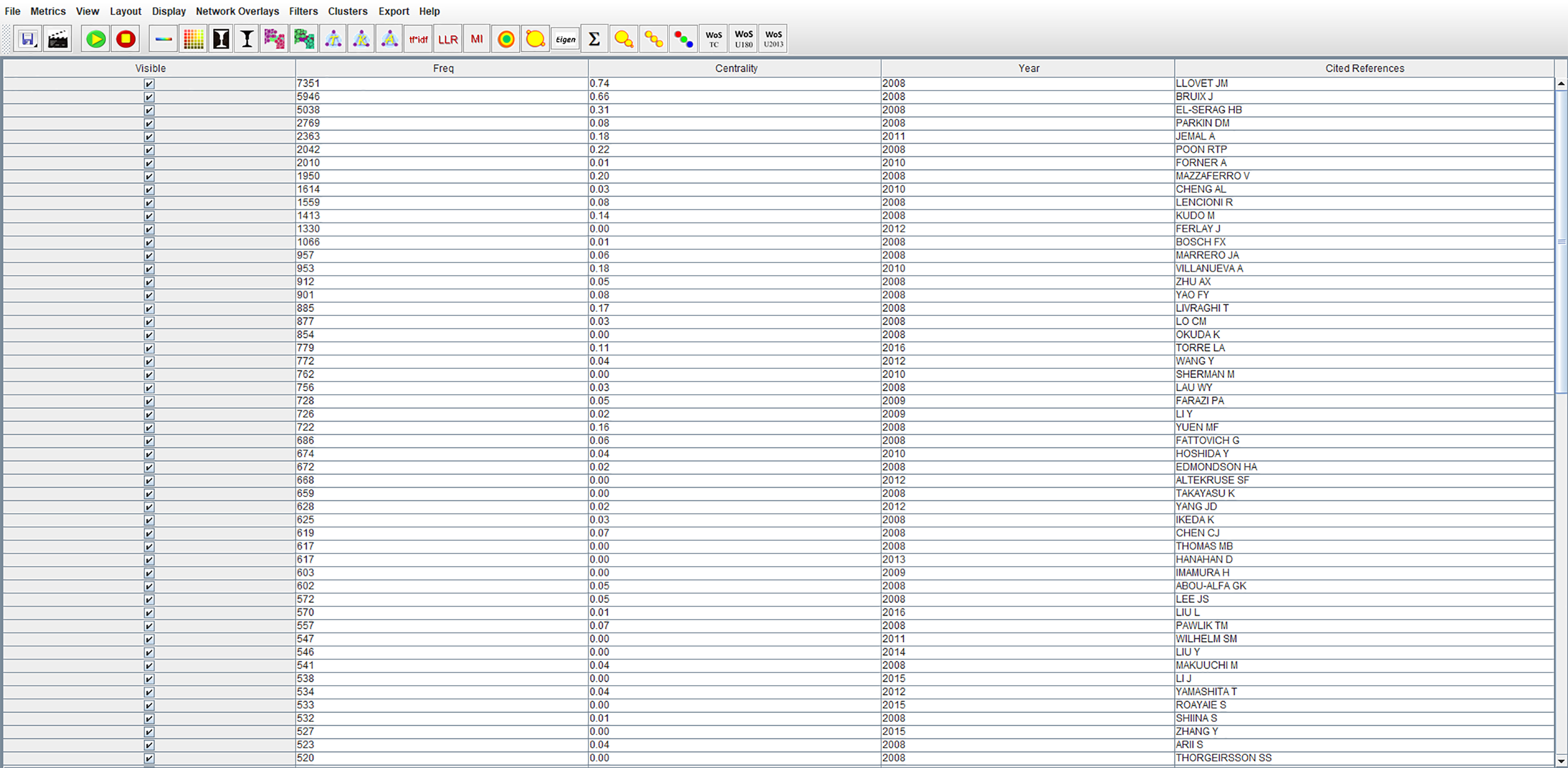

Supplement: Figure S2 [file peerj-06-5477-s006.png]

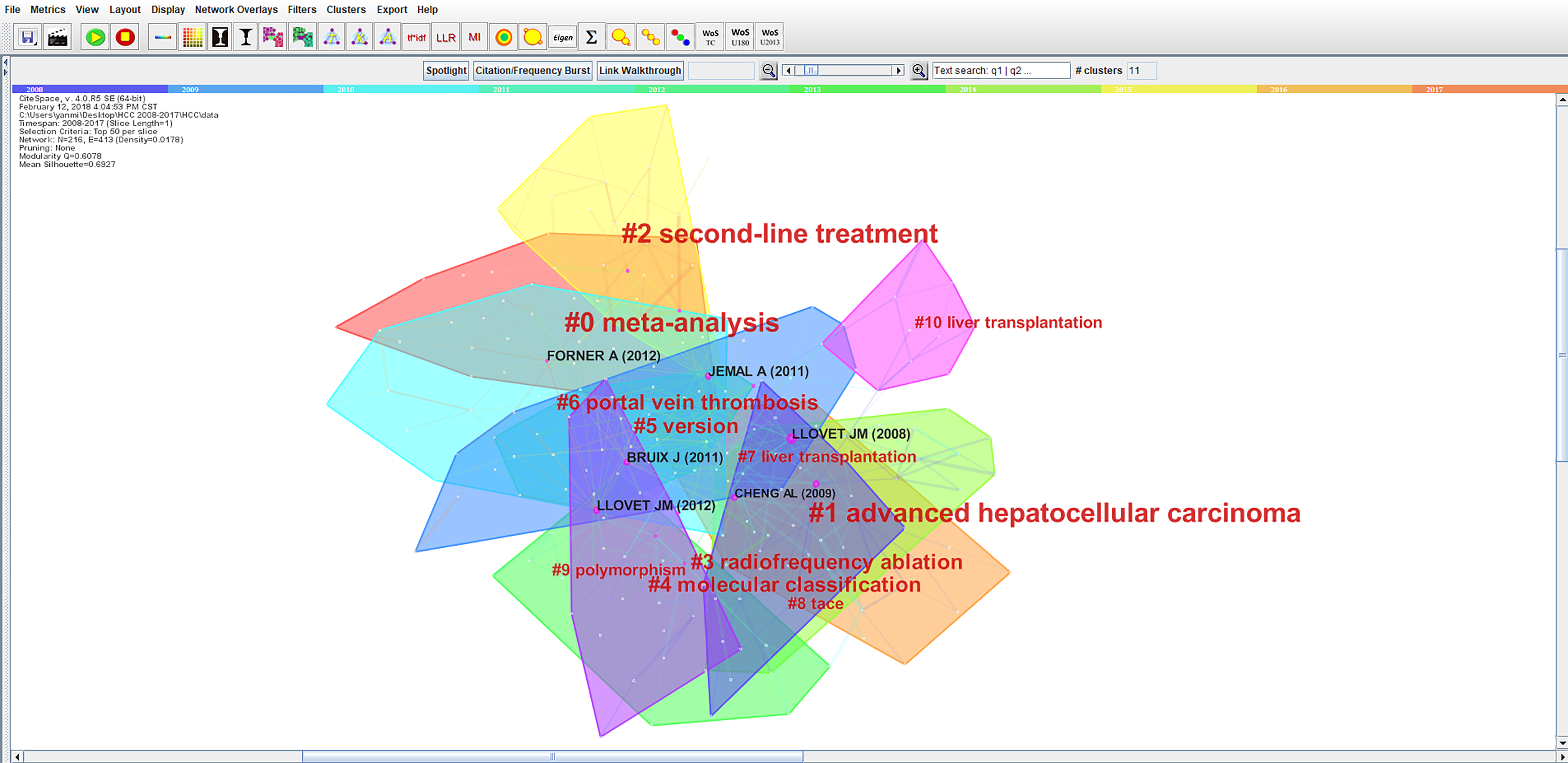

Supplement: Figure S3 [file peerj-06-5477-s007.png]

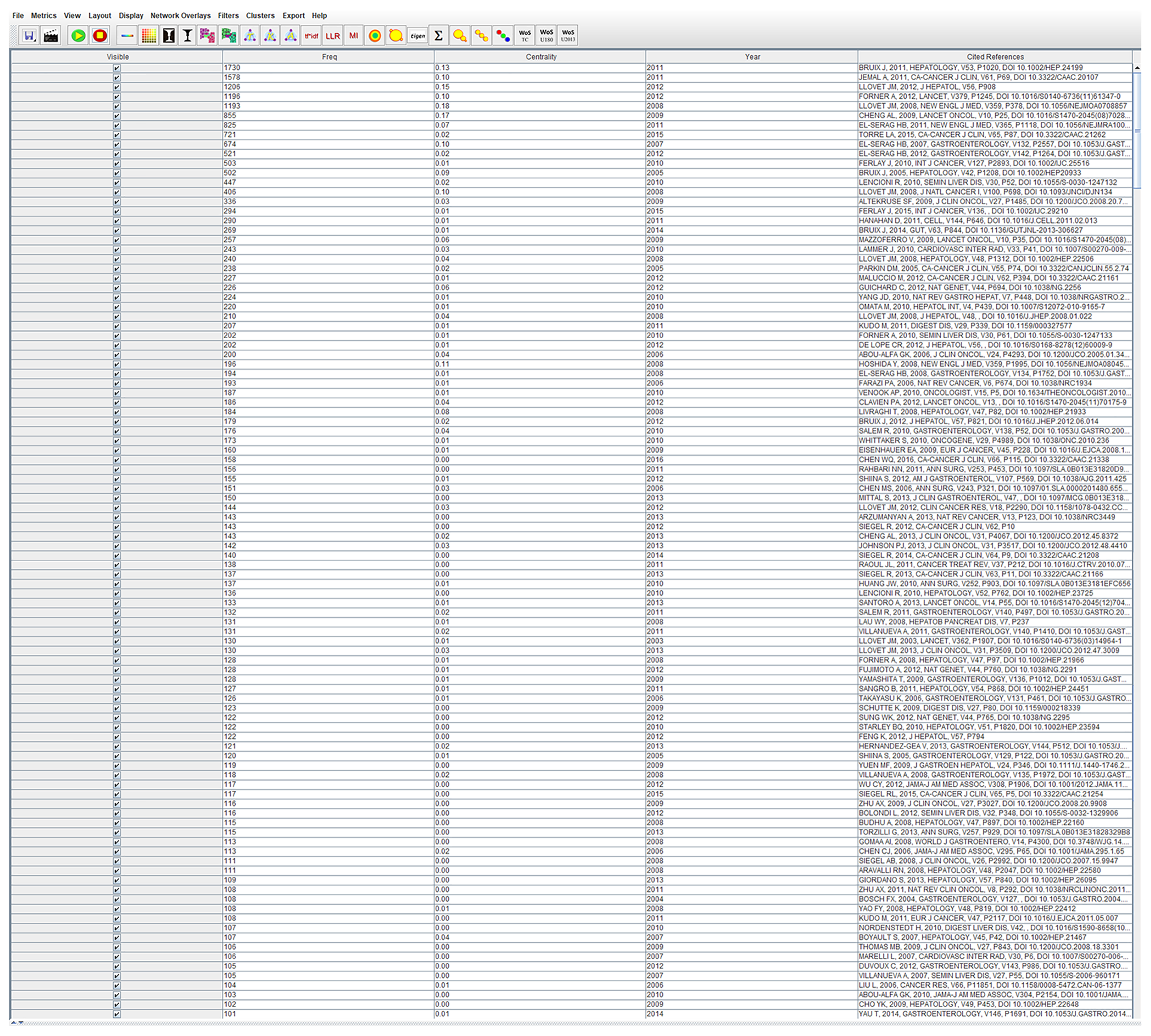

Supplement: Figure S4 [file peerj-06-5477-s008.png]

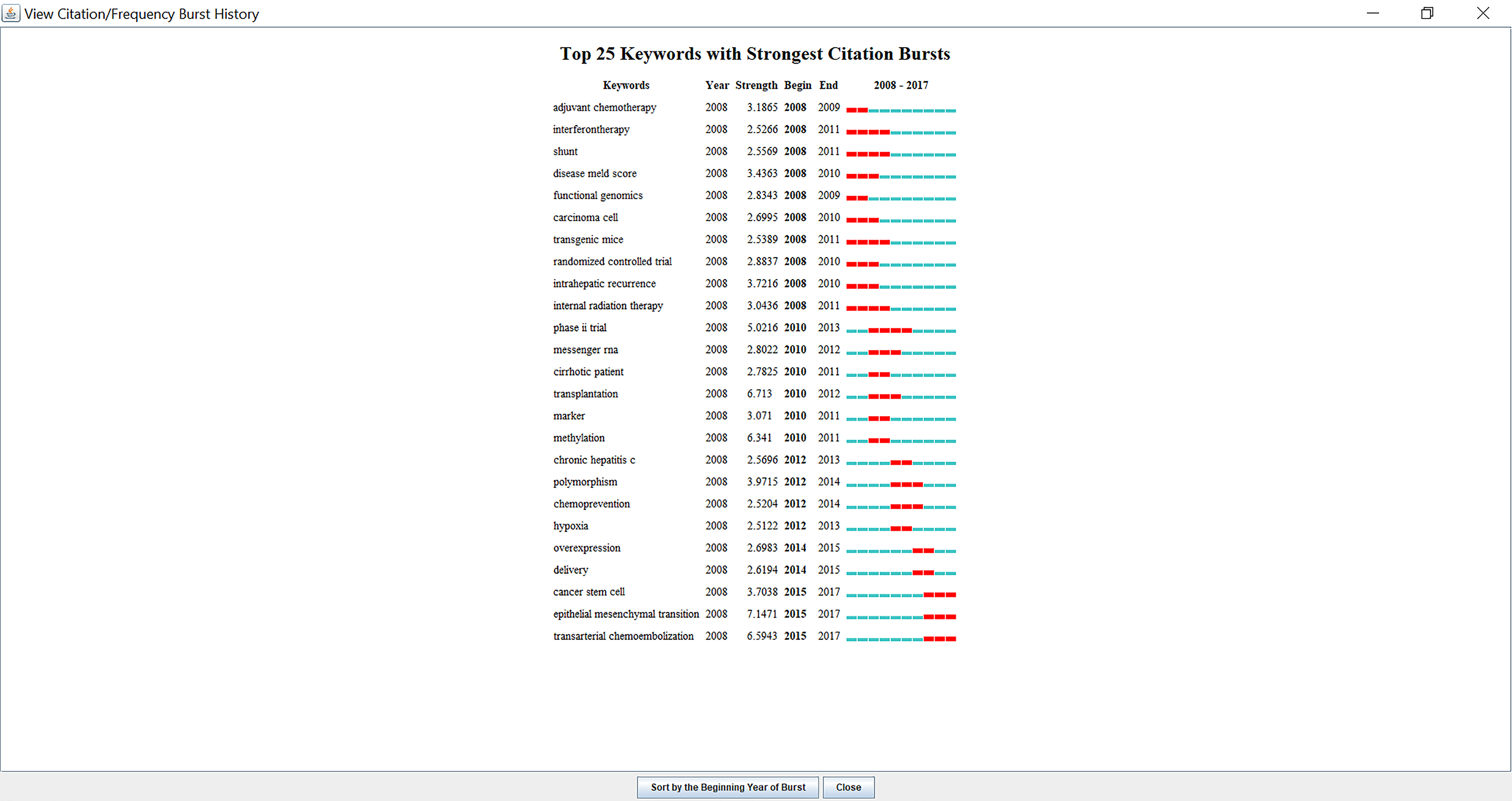

Supplement: Figure S5 [file peerj-06-5477-s009.png]

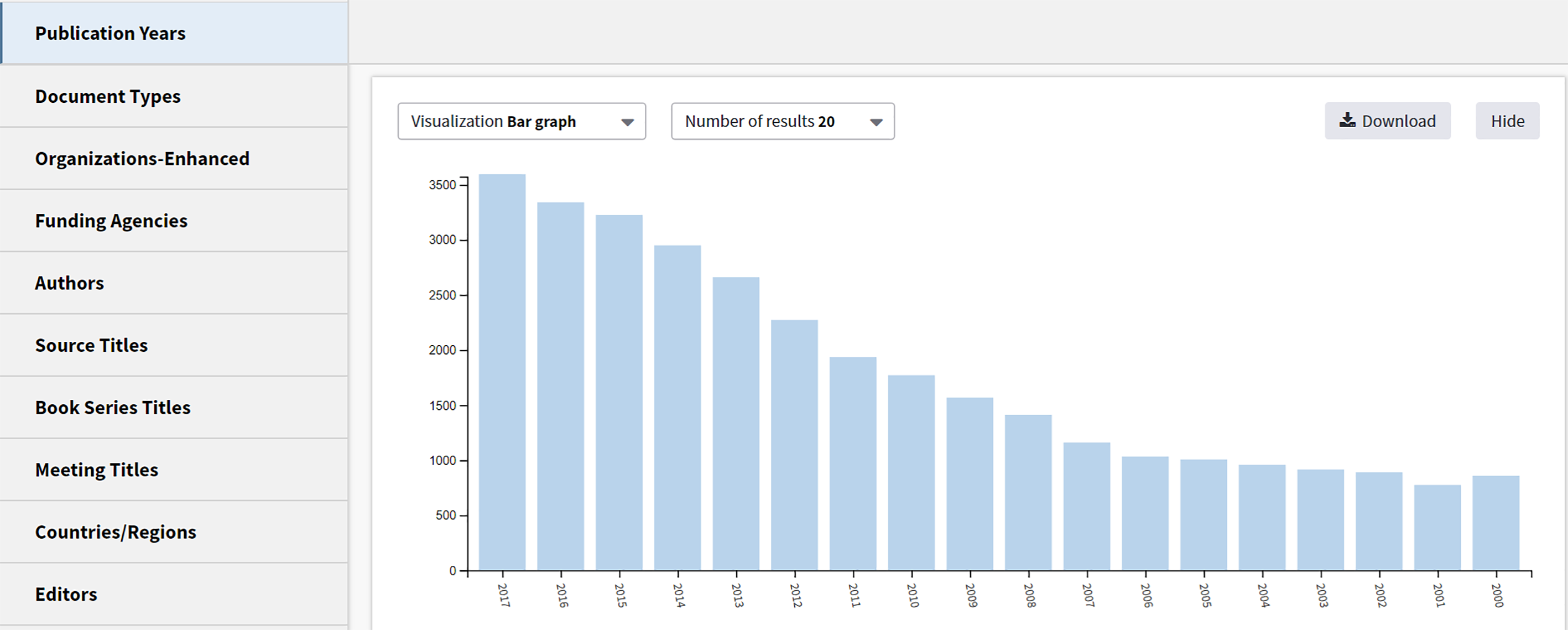

Supplement: Figure S6 [file peerj-06-5477-s010.png]
